# Visualization of Inferior Alveolar and Lingual Nerve Pathology by 3D Double-Echo Steady-State MRI: Two Case Reports with Literature Review

**DOI:** 10.3390/jimaging8030075

**Published:** 2022-03-17

**Authors:** Adib Al-Haj Husain, Daphne Schönegg, Silvio Valdec, Bernd Stadlinger, Thomas Gander, Harald Essig, Marco Piccirelli, Sebastian Winklhofer

**Affiliations:** 1 Clinic of Cranio-Maxillofacial and Oral Surgery, Center of Dental Medicine, University of Zurich, 8032 Zurich, Switzerland; adib.al-hajhusain@uzh.ch (A.A.-H.H.); silvio.valdec@zzm.uzh.ch (S.V.); bernd.stadlinger@zzm.uzh.ch (B.S.); 2 Clinic of Cranio-Maxillofacial and Oral Surgery, University Hospital of Zurich, University of Zurich, 8091 Zurich, Switzerland; daphne.schoenegg@usz.ch (D.S.); thomas.gander@usz.ch (T.G.); harald.essig@usz.ch (H.E.); 3 Department of Neuroradiology, Clinical Neuroscience Center, University Hospital of Zurich, University of Zurich, 8091 Zurich, Switzerland; marco.piccirelli@usz.ch

**Keywords:** magnetic resonance imaging, third molar surgery, nerve injury, inferior alveolar nerve, lingual nerve, oral surgery, oral radiology, oral anatomy

## Abstract

Injury to the peripheral branches of the trigeminal nerve, particularly the lingual nerve (LN) and the inferior alveolar nerve (IAN), is a rare but serious complication that can occur during oral and maxillofacial surgery. Mandibular third molar surgery, one of the most common surgical procedures in dentistry, is most often associated with such a nerve injury. Proper preoperative radiologic assessment is hence key to avoiding neurosensory dysfunction. In addition to the well-established conventional X-ray-based imaging modalities, such as panoramic radiography and cone-beam computed tomography, radiation-free magnetic resonance imaging (MRI) with the recently introduced black-bone MRI sequences offers the possibility to simultaneously visualize osseous structures and neural tissue in the oral cavity with high spatial resolution and excellent soft-tissue contrast. Fortunately, most LN and IAN injuries recover spontaneously within six months. However, permanent damage may cause significant loss of quality of life for affected patients. Therefore, therapy should be initiated early in indicated cases, despite the inconsistency in the literature regarding the therapeutic time window. In this report, we present the visualization of two cases of nerve pathology using 3D double-echo steady-state MRI and evaluate evidence-based decision-making for iatrogenic nerve injury regarding a wait-and-see strategy, conservative drug treatment, or surgical re-intervention.

## 1. Introduction

The trigeminal nerve, the fifth cranial nerve (CN V), is one of the largest peripheral sensory nerves and provides sensory innervation to the face, eyes, mouth, and scalp via its major branches (ophthalmic (V1), maxillary (V2), and mandibular (V3)) [1]. During invasive dental procedures or oral and maxillofacial surgery, the mandibular nerve, the only branch of the CN V containing a motor root, is at risk of peripheral iatrogenic damage [2]. Mandibular third molar (MTM) surgery, one of the most common surgical procedures in dentistry and also the most commonly associated with nerve injury, can cause iatrogenic nerve damage predominantly to the lingual nerve (LN) in the lingual posterior mandibular region or the inferior alveolar nerve (IAN) either in its osseous mandibular canal or prior to its entry into the oval foramen [3,4]. Regarding the incidence of MTM surgery-associated nerve injuries, there are heterogeneous data in the literature, with IAN injuries estimated at approximately 4% [3,4,5] and LN injuries at about 1–2% [6]. Fortunately, most LN and IAN injuries recover spontaneously within three to six months. Longer-lasting sensory disturbances with no signs of recovery within this time frame may remain permanent and result in a significant loss of quality of life for the affected patients [7]. Once nerve damage has occurred, most patients often suffer from additional neuropathic pain apart from anesthesia, hypesthesia, or paresthesia in the area supplied by the nerve [8].

Therefore, a thorough preoperative radiologic risk assessment of the surgical site, especially the location of peripheral nerves in MTM surgery, is key to avoiding these unpleasant postoperative complications. Panoramic radiography (PAN), which provides a two-dimensional overview of the anatomy and location of the MTM and surrounding anatomical structures, is routinely obtained as the initial diagnostic modality. However, in cases displaying a close positional relationship between the roots of the MTM and the mandibular canal, discontinuous cortical delineation or diversion of the mandibular canal, PAN is not sufficient, and three-dimensional information of the region of interest is often required [9]. Cone-beam computed tomography (CBCT), currently considered the “gold standard” for this medical task, provides three-dimensional cross-sectional images of the relevant anatomical structures in the MTM region, including the number and shape of the MTM roots and the buccolingual localization and cortical integrity of the mandibular canal [10].

However, conventional radiographic imaging techniques can only visualize the IAN indirectly via the osseous boundaries of the mandibular canal, whereas the nervous tissue of the IAN and LN per se cannot be depicted directly. For this purpose, magnetic resonance imaging (MRI), with the recently introduced black-bone MRI sequences, can simultaneously visualize osseous structures and neural tissue in the oral cavity with high spatial resolution and excellent soft-tissue contrast [11,12,13,14]. Although most LN and IAN injuries recover spontaneously, therapy should be initiated early in selected cases with evident nerve damage, although the literature is inconsistent regarding the therapeutic time window [15].

Therefore, in this report, we present two cases where visualization of nerve pathology using 3D double-echo steady-state (DESS) MRI supported clinical decision-making. In addition, we reviewed the current literature to evaluate evidence-based decision-making regarding a wait-and-see strategy, conservative drug treatment, or surgical re-intervention.

## 2. Case Presentation

### 2.1. Case 1

A 70-year-old female patient presented to the Clinic of Cranio-Maxillofacial and Oral Surgery complaining of constant numbness in the left cheek, chin, and mandible, partly with radiation to the ear and temporal area for several weeks, without exhibiting pain or impairment of masticatory function. Clinical findings showed tingling on palpation of the jaw, no signs of inflammation, and occlusion and mouth opening without abnormalities. The remaining sensorimotor function of cranial nerves V and VII showed no pathological findings. An MRI scan was performed to rule out mandibular nerve pathology and osteomyelitis, using conventional imaging protocols and the DESS sequence, which is specifically suited for peripheral nerve imaging (Table 1). The conventional MRI protocol included native T2 and T1 Turbo Spin-Echo (TSE) 2D acquisitions in axial and coronal orientations, a diffusion weighted segmented echo planar imaging, and post contrast agent injection T1 TSE fat saturated axial, coronal, and sagittal acquisitions.

**Figure 1:**
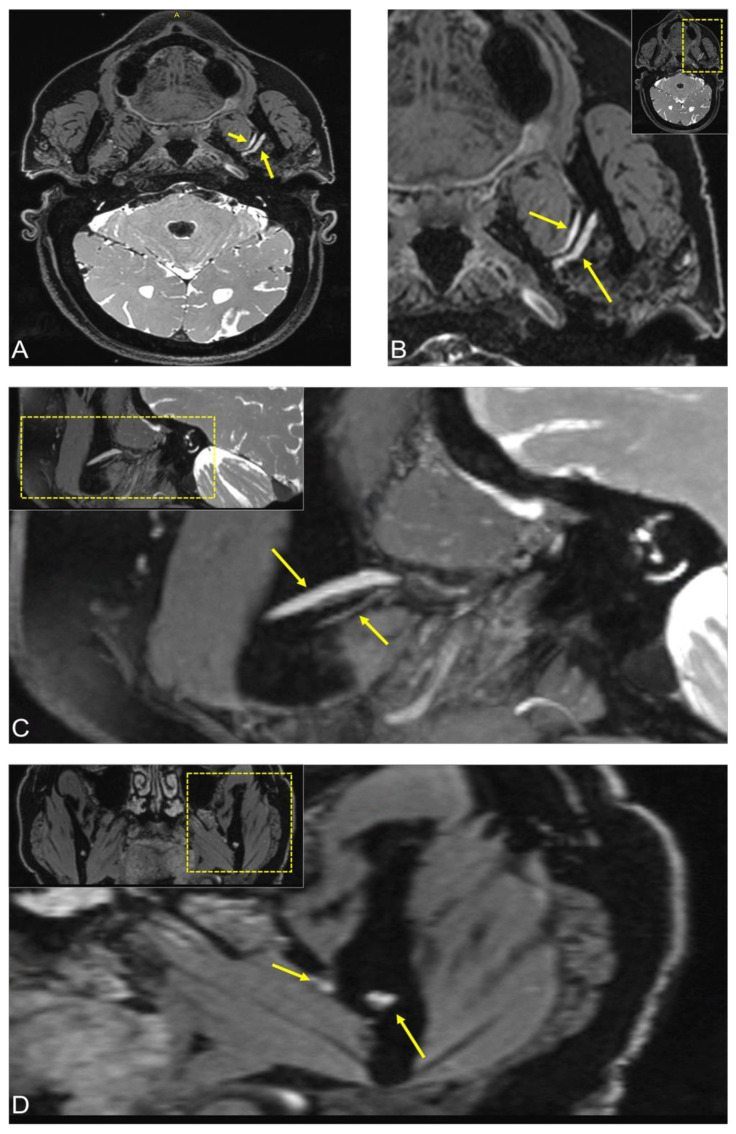
3D Double-Echo Steady State (DESS) MRI of a patient complaining of constant numbness in the left cheek, chin, and mandible, partly with radiation to the ear and temporal area. The DESS protocol showed osseous signal alteration on the left mandible in the third quadrant in the molar region. (**A**,**B**) Axial, (**C**) sagittal, and (**D**) coronal side-by-side comparison of the image reconstructions showed hyperintense signal alterations of the mandibular nerve from the oval foramen through its branches in the inferior alveolar nerve and lingual nerve to the region of the third molar. In all images, the short arrow shows signal hyperintensities of the lingual nerve at the anatomic point where it begins to pass from lateral to medial, and the longer arrow represents the inferior alveolar nerve before it enters into the mandibular foramen. For orientation, the dotted rectangles in the corner show the enlarged area.

MRI revealed bilateral advanced temporomandibular joint arthrosis with left effusion and synovial contrast enhancement. The DESS sequence showed osseous signal alteration on the left mandible in the third quadrant in the molar region. In addition, the side-by-side comparison showed hyperintense signal alterations of the mandibular nerve from the oval foramen through its branches in the IAN and LN to the region of the third molar. The pathology’s exact cause remained unclear, but imaging ruled out space-occupying lesions and revealed precise localization of the pathologic process of the nerves with a suspected acute inflammatory process (Figure 1).

The neurological stimulation with sensory evoked potentials of the lower lip showed regular trigeminal potentials on both sides with normal latencies (21.1 ms; norm < 22.3 ms). In addition, the neurological status was without pathological findings, and multidetector computed tomography (CT) of the thorax excluded sarcoidosis. After completion of all investigations, which ruled out any systemic disease, the patient was prescribed off-label therapy with prednisone as empirical treatment of suspected inflammation of the mandibular nerve. 

**Figure 2:**
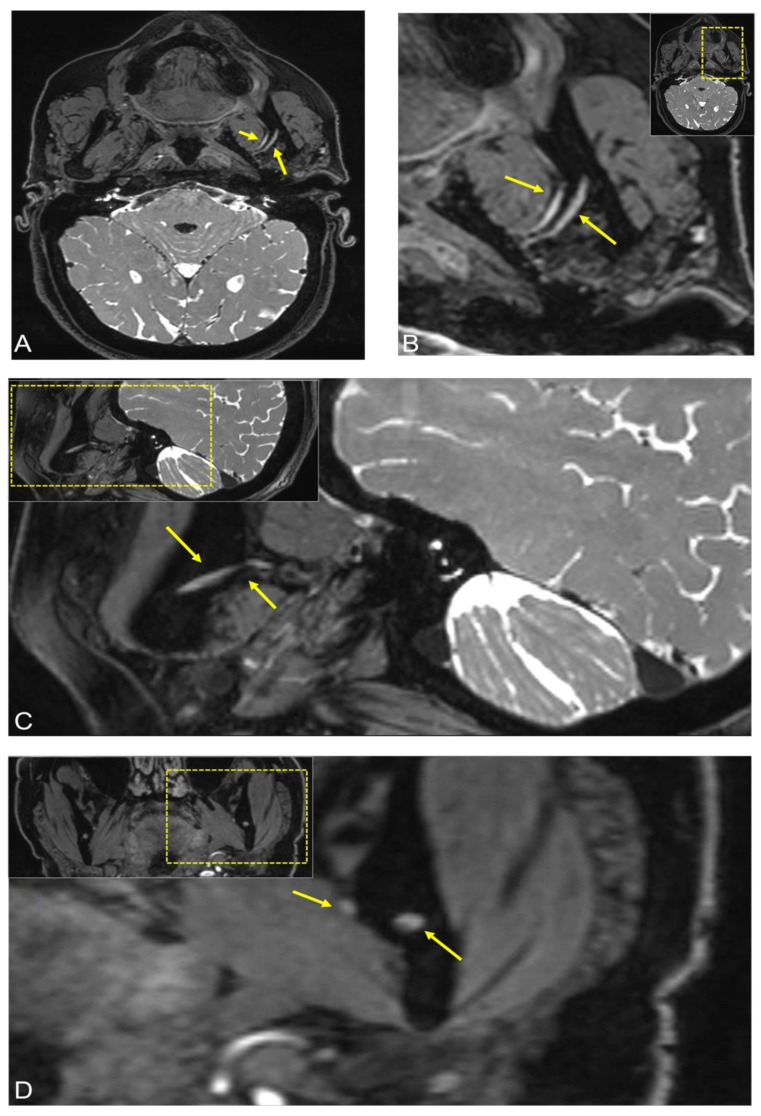
Follow-up MRI after three months of the same patient. The 3D Double-Echo Steady State (DESS) MRI protocol revealed the following: (**A**,**B**) Axial, (**C**) sagittal, and (**D**) coronal side-by-side comparison of the image reconstructions showed bilateral symmetry of the mandibular nerve with a loss of signal intensity in the 3D-DESS sequence compared with the previous examination, no longer indicating evident pathological signal alterations. In all images, the short arrow shows signal hyperintensities of the lingual nerve at the anatomic point where it begins to pass from lateral to medial and the longer arrow represents the inferior alveolar nerve before it enters into the mandibular foramen. For orientation, the dotted rectangles in the corner show the enlarged area.

After three months, the incipient regredience of the inflammation could be confirmed clinically, as the patient stated that the numbness had subsided and only occurred sporadically for a short time, although she had independently discontinued the prednisone therapy after ten days. Follow-up MRI showed bilateral stationary temporomandibular joint arthrosis with marked flattening of the mandibular condyles and a stationary small joint effusion on the left side with synovial contrast enhancement. Mildly hyperintense signal alterations were still visible in the third quadrant in the molar region in the DESS sequence and the contrast-enhanced fat-saturated T1-weighted sequence. The mandibular nerve, especially the IAN and the LN, now showed bilateral symmetry with a loss of signal intensity in the DESS sequence compared with the previous examination, no longer indicating evident pathological signal alterations (Figure 2). 

Consequently, treatment was terminated, and a follow-up MRI was scheduled in six months. 

### 2.2. Case 2

**Figure 3:**
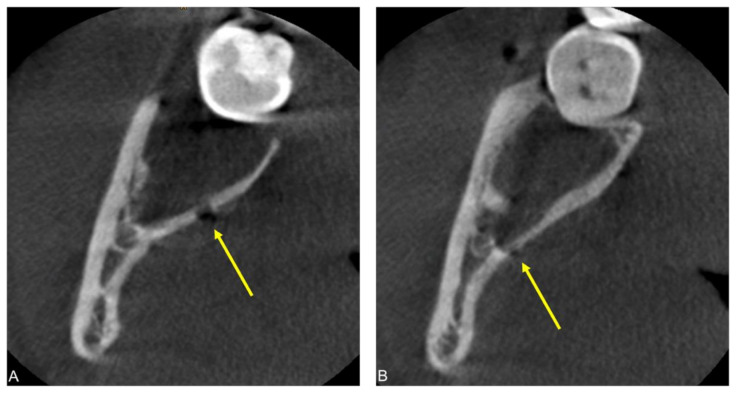
Postoperative cone-beam computed tomography (CBCT) scan of a patient complaining of persistent numbness of the right side of the tongue two weeks after bilateral MTM surgery. Axial CBCT image reconstructions (**A**,**B**) visualize two possible iatrogenic defects of the lingual cortical bone: a spherical lesion at the level of the former crown and a linear defect distal to the level of the former cementoenamel junction of the MTM.

An 18-year-old female patient presented to the Clinic of Cranio-Maxillofacial and Oral Surgery two weeks after bilateral MTM surgery, complaining of persistent numbness of the right side of the tongue. The patient recalled non-electrifying pain upon injection of the local anesthesia on the right side. Additionally, the patient reported pain at the base of the tongue on the right side triggered by cold beverages or accidental tongue biting. Clinical exam proved total anesthesia of the right side of the tongue regarding the perception of pain, touch, and temperature, as well as an impaired taste. The tongue showed discrete atrophy of the papillae on the right side. The healing process was otherwise uneventful without signs of inflammation and the remaining sensorimotor function of cranial nerves V and VII were intact. A postoperative CBCT scan was performed showing two possible iatrogenic defects of the lingual cortical bone suggestive of bone injury with a bur during ostectomy: a spherical lesion at the level of the former crown and a linear defect distal to the level of the former cementoenamel junction of the MTM (Figure 3).

**Figure 4:**
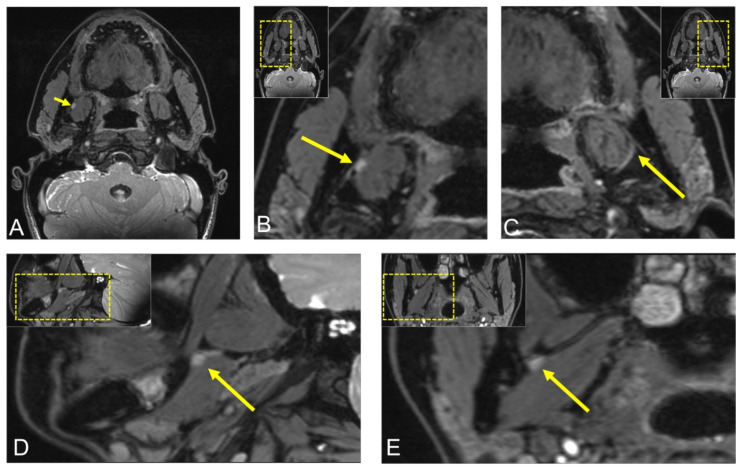
Postoperative visualization of the lingual nerve (LN) using 3D double-echo steady-state (3D-DESS) MRI in the same patient. (**A**,**B**) Axial, (**D**) sagittal, (**E**) coronal reconstruction of the region of interest displaying the LN asymmetry throughout its course through the infratemporal fossa, with an LN hyperintense nodular lesion of 5 × 3 mm between the right medial pterygoid muscle and the mandible. (**C**) Axial image reconstruction of the healthy LN on the contralateral side for comparison. For orientation, the dotted rectangles in the corner show the enlarged area.

An MRI scan, including the DESS MRI protocol, was performed to visualize the lingual nerve up to its terminal branches and evaluate whether radiologically visible damage to the LN could be detected. In the DESS sequence, the LN asymmetry is visible throughout its course through the infratemporal fossa, with an LN hyperintense and T1-contrast-enhancing nodular lesion of 5 × 3 mm between the right medial pterygoid muscle and the mandible, consistent with a neuroma. Visualization of the LN at the level of the osteotomy was complex in this case, as T1-weighted imaging did not show reliable signal alterations, whereas the 3D-DESS sequence demonstrated hyperintense signal alterations. However, no complete separation of the lingual nerve could be detected (Figure 4). Fusion of postoperative CBCT with MRI confirmed this information, with these signal alterations precisely localized at the level of the longitudinal cortical perforation. 

Considering that the LN did not appear to be transected in the MRI and that the patient had started to regain sensitivity to touch and temperature less than three months postoperatively, a wait-and-see approach was suggested. Thorough clinical aftercare of the patient is planned, with radiological follow-up scheduled in a few months. 

## 3. Discussion

State-of-the-art surgical treatment strategies in MTM surgery strive for low-risk, minimally invasive, hard-tissue preserving interventions. In this regard, a multimodal surgical planning considering the medical record, preoperative radiographic imaging, and intraoperative features of the MTM region is key to reducing perioperative risks [16]. Nevertheless, iatrogenic nerve injuries are rare fateful events with severe consequences for affected patients, which cannot be fully avoided in each complex surgical procedure with today’s standard preoperative imaging modalities. Consequently, an early planning of further therapy within the therapeutic time window is crucial in case of nerve damage. For this reason, this review article highlights and presents the current clinical treatment and diagnostic imaging options. 

While conventional radiographic imaging techniques such as PAN or CBCT can provide anatomical information about the region of interest—such as the position, depth, and angulation of the MTM, the surrounding bony borders of the radiolucent mandibular canal, the maxillary sinuses, and the presence of pathologic processes—the direct visualization of the IAN and LN is not possible [16]. Given the recent improvements in MRI, a radiation-free cross-sectional imaging technique, and the development of new MRI protocols suitable for dental imaging, it is nowadays possible to provide detailed anatomical images of the oral cavity and its contents with high spatial resolution and excellent soft-tissue contrast [14,17,18]. Older MRI studies on this topic used conventional, nonspecific MRI protocols with a field strength of 1 Tesla, resulting in a low signal-to-noise ratio that can be considered insufficient and limiting from today’s standpoint. However, it was possible to depict the neurovascular bundle in the MTM region as a moderately hyperintense signal without distinguishing the neural tissue from the blood vessels [19,20]. Meanwhile, thanks to the use of higher field strengths of 3 Tesla, intraoral or radiofrequency coils, and specific dental MRI protocols, the quality of imaging has significantly improved, and promising visualization has been achieved [21,22].

The increasing number of MRI studies that have used black bone MRI sequences in recent years illustrates their great potential for various dental applications. In this context, new developments target novel MRI protocols modified for dental applications, such as 3D-DESS and 3D short-tau inversion recovery (STIR), which enable high-resolution and high-contrast reliable assessment of the MTM region [23]. In addition to simultaneous visualization of bone and soft tissues, it is possible to visualize the positional relationship between the IAN/LN and MTM [18], to precisely determine the localization of the IAN within the osseous boundaries of the mandibular canal [14], and to visualize the lingual nerve up to its insertion into the tongue both focally and continuously [12]. In these dedicated black bone MRI protocols, the free induction decay (FID) signals of fast imaging with steady-state precession (FISP) are combined with the echo signal of a time-reversed FISP (PSIF), reducing signal dropout due to dephasing while simultaneously increasing its T2* specificity. Therefore, peripheral nerve branches such as the IAN and LN are depicted as high signal intensity structures and can be distinguished from the adjacent anatomical structures by applying the water excitation/fat suppression technique due to the myelin layer surrounding the nerves [24]. When comparing different black bone MRI sequences and their application in the dental field, Burian et al. reconfirmed that they are best suited for this medical task [23]. The STIR protocol provided the best signal-to-noise ratio and the best nerve-muscle contrast-to-noise ratio, while the DESS protocols were best for comparing quantitative parameters [23]. Even in more complex medical conditions, especially in space-occupying lesions, the contrast-enhanced 3D SPACE STIR sequence demonstrated accurate localization of the morphological changes of the peripheral nerve branches and the associated pathological process with higher nerve tissue contrast compared with conventional MR neurography sequences [25]. As demonstrated in the case reports of this review article, these imaging protocols can be used in postoperative follow-up and the evaluation of ambiguous nerve pathology of the IAN and LN. This additional data could provide useful supplemental information to assess what type of nerve damage is present, leading to improved strategies for perioperative management and thus, if necessary, facilitating preoperative planning for possible secondary nerve reconstruction. 

The major difficulties with MRI of the oral cavity remain motion artifacts, field inhomogeneity, and metallic dental restorations causing artifacts [26]. However, specific modifications for artifact suppression are already established but need to be further improved [27].

Injury to the IAN and LN during and after MTM surgery can have a variety of causes, including direct trauma to the nerve from rotating instruments during ostectomy or separation, sharp transection during flap preparation, or more indirect damage from compression or chemical disturbance from a hematoma or injection of local anesthetics [28,29,30,31]. Apart from complete transection of a nerve, which is usually noticed immediately during surgery, it is often difficult to determine the exact cause of nerve injury. Diagnostics include clinical assessment of nerve function, neurophysiological testing, and, nowadays, MRI imaging. 

If almost complete or even complete transection of the IAN or LN is suspected, immediate exploration and microsurgical reconstruction of the nerve is indicated. Surgical techniques include direct suturing, insertion of an interposition graft, or nerve conduits, among others [32,33,34].

The literature is inconsistent regarding the optimal treatment of less severe cases. Conservative treatment with a wait-and-see approach might be justified if patients are not disturbed by decreased sensibility or neuropathic pain. Pharmacologic treatment with a vitamin B complex or steroids might support nerve healing [35,36,37]. Fortunately, spontaneous recovery—albeit slow—is likely. Close clinical follow-up is needed to monitor progress. In symptomatic patients who do not show signs of improvement over time, surgical exploration and, if needed, intervention must be discussed. The overall outcome, as measured by functional sensory recovery, is acceptable, and quality of life can often be increased [31]. While some authors recommend waiting 90 days for spontaneous healing, recent research suggests improved outcomes after early intervention [38,39]. Significant subjective and objective improvement in both patients presented in the case report confirms the conservative approach chosen.

Considering the steadily increasing number of MRI studies in dentistry and specifically oral and maxillofacial surgery, and the associated diverse and steadily increasing demands for high-resolution perioperative noninvasive imaging modalities, it is necessary to evaluate the evidence of added benefits as well as the indications and limitations of black bone MRI sequences in further prospective cohort studies for this specific medical task and thus provide evidence-based recommendations for optimized decision-making. 

## 4. Conclusions

Black-bone MRI protocols are another step towards personalized dentistry and improved decision-making. They have the potential to prevent ineffectiveness and reduce perioperative risks in oral surgery by considering additional patient-related parameters such as anatomical aberrations and imaging biomarkers. However, PAN and CBCT will remain the primary diagnostic tools of choice, while the targeted use of dental MRI could improve patient management through adequate risk assessment and improved postoperative care with an early referral for potential nerve injury, leading to better patient outcomes.

## Figures and Tables

**Table 1:** Main technical parameters of the 3D Double-Echo Steady-State (DESS) MRI protocol.

Double-Echo Steady-State (DESS) MRI Parameters	
Acquisition time	12:24 min:s
Field of View (FOV)	242 × 242 × 78 mm^3^
Acquisition matrix	320 × 320 × 104
Acquisition voxel	0.75 × 0.75 × 0.75 mm^3^
Number of signal averages	1
Repetition Time (TR)	11.2 ms
Time to Echo 1 (TE1)	4.2 ms
Time to Echo 2 (TE2)	7.7 ms
WFS (pix)/bandwidth (Hz/Px)	1/355
Fat suppression	Selective water excitation
Parallel acquisition	No

## Data Availability

The complete data presented in this study are available on request from the corresponding author. The data are not publicly available due to privacy restrictions.
